# Factors Influencing User Satisfaction with Information Systems: A Systematic Review

**DOI:** 10.31661/gmj.v9i0.1686

**Published:** 2020-06-26

**Authors:** Leila R Kalankesh, Zahra Nasiry, Rebecca A Fein, Shahla Damanabi

**Affiliations:** ^1^School of Management and Medical Informatics, Tabriz University of Medical Sciences, Tabriz, Iran; ^2^Health Services Management Research Center, Tabriz University of Medical Sciences, Tabriz, Iran; ^3^A.T. Still University, College of Graduate Health Studies, Missouri, USA

**Keywords:** Information System, User, Satisfaction, Multivariate Analysis

## Abstract

User satisfaction has been considered as the measure of information system effectiveness success. User satisfaction is difficult to define but is considered an evaluation construct. Globally health organizations, particularly hospitals, invest a huge amount of money on information system projects. If hospital information systems (HISs) are to be successful, factors influencing or related to user satisfaction should be taken into account at the time of designing, developing or adopting such systems. The current study aimed to provide a comprehensive review of factors related to user satisfaction with information systems. The researchers systematically searched PubMed, Science Direct, and IEEE electronic databases for articles published from January 1990 to June 2016. A search strategy was developed using a combination of the following keywords: "model," "user satisfaction," "information system," "measurement," "instrument," and " tool." Reported dimensions, factors, and their possible influence on user satisfaction with information systems were extracted from the studies wherever was possible. Overall factors influencing user satisfaction with information systems can be categorized in seven dimensions: Information quality, system quality, vendor support quality, system use, perceived usefulness, user characteristics, and organizational structure & management style. If all these factors are considered properly in the process of developing, designing, implementing, or purchasing information systems, the higher user satisfaction with the system will be likely. Otherwise, it would end up with unsatisfied users that will finally contribute to the system failure.

## Introduction

One measurement of information system success is user satisfaction [1,2]. User satisfaction is intangible; therefore, it lacks an objective definition [3]. Globally health care organizations, particularly hospitals, invest a huge amount of money on information system projects, and user satisfaction is vital to successful implementations [2]. In some cases, user satisfaction is the only factor that determines if an information system’s performance is acceptable to the organization [4]. If the information system is to be successful, factors influencing or related to user satisfaction should be taken into account at the time of designing, developing, or adopting such systems [2]. The current study aimed to characterize different dimension and their related factors that have been considered as influencing factors of user satisfaction with the information system.

## Search Strategies

**Figure 1:**
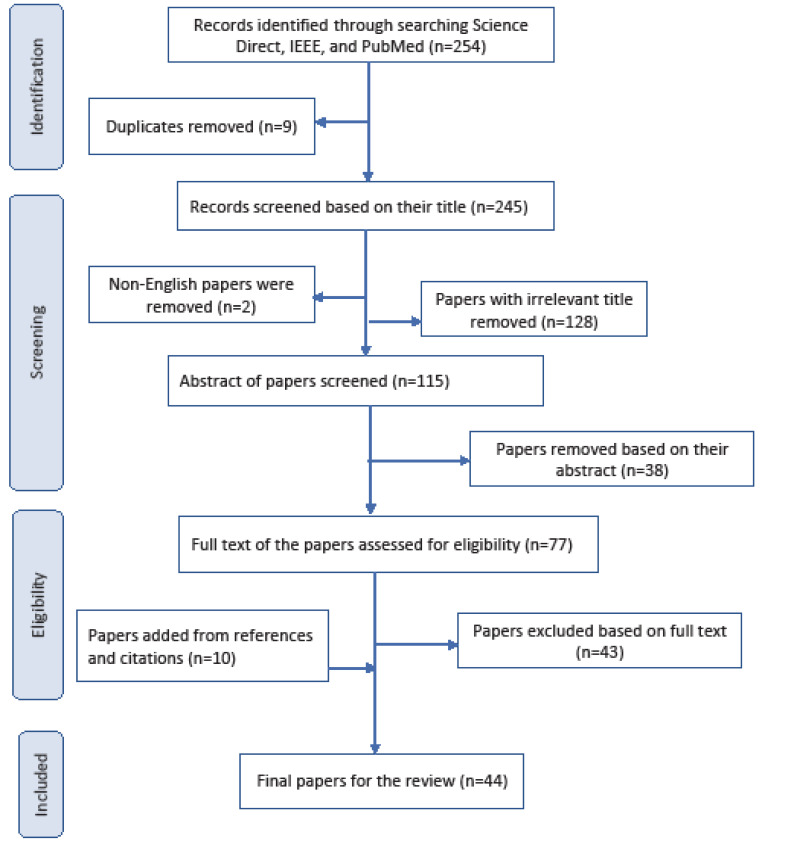
PRISMA flowchart representing the selection of studies for the systematic review of the factors influencing user satisfaction with information systems

A systematic literature review was carried out on PubMed, Science Direct, and IEEE electronic databases for articles published in the last 26 years (from January 1990 to June 2016). A search strategy was developed using a combination of the following keywords: “model,” “user satisfaction,” “information system,” “measurement,” “instrument,” and “tool.” Figure-1 shows PRISMA diagram for the process of selecting studies for the detailed review. Studies were included if they reported factors related to user satisfaction with information systems. After screening and examining the retrieved papers, 44 studies [2,5-47] were found qualified to be included in data extraction.

## Results

###  Dimensions of User Satisfaction 

**Figure 2:**
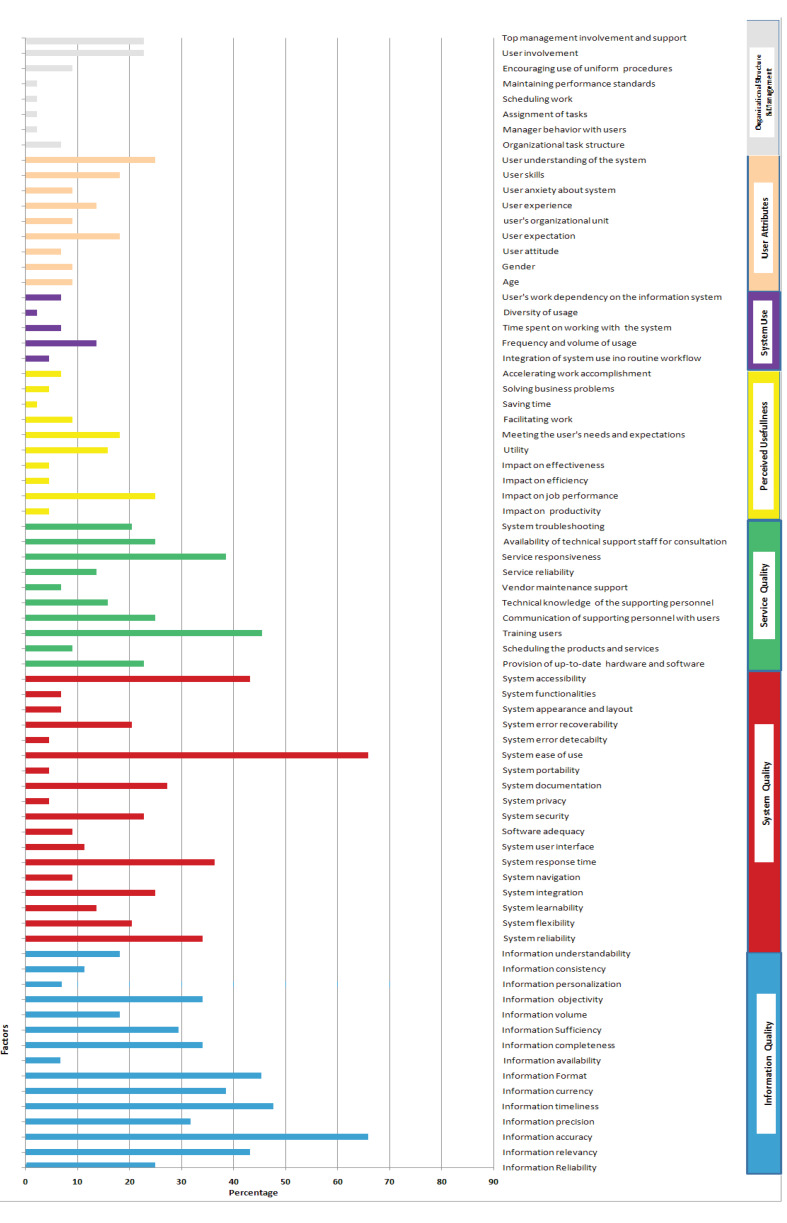
Percentage of factors influencing user satisfaction with information systems

 As Figure-2 illustrates factors presented as influencing the user satisfaction with information systems could be categorized into seven dimensions include information quality, sy tem quality, service or vendor support quality, system use, perceived usefu ness, user characteristics, organizational structure, and management style. Dimensions of system quality and information quality have the highest number of factors (18 and 15 factors respectively), followed by vendor support quality. In comparing these three dimensions with the other four, there is significantly more influence by these three factors over the other four. Factor loading indicates to some extent the factor effect weight on user satisfaction. The majority of the factor loadings have been reported in the domain of information quality followed by system quality.

###  Information Quality

Information quality has several attributes reliability, relevancy, accuracy, precision, timeliness, currency, format, availability, completeness, sufficiency, volume, objectivity, perso alization, consistency, and understandability. Information accuracy was mentioned as the most prevalent (about 65%) attribute in the studies. Information timeliness was highligh ed in about 47.7% of the studies followed by information format (45.5%) and information relevancy (43.2%). [2,5,7-16,18-20,23-27,31,32,34-36,39-47].

###  System Quality

Attributes of system quality include system reliability, system flexibility, system learnability, system integration, system navigation, system response time, system user interface, software adequacy, system security, system privacy, system documentation, system portability, system ease of use, system error delectability, system error recoverability, system appearance and layout, system functionality, and system accessibility. System ease of use (65.9 %), system accessibility (43.18%), system response time (36.4%), and system reliability (34.1%) were top four most prevalent attributes considered in the models [2,5,7-16,18-20,22-24,26,27,29-47].

###  Service Quality

A total number of 10 factors were identified for service quality or vendor support quality. Factors highlighted in this dimension range from provision of up-to-date software and hardware for information system to scheduling products and services, training users, communication of supporting staff with users, technical knowledge of the supporting staff, vendor or developer maintenance support, service reliability, service responsiveness, availability of supporting staff, and system troubleshooting. Among these factors, training users was the most prevalent factor in the models (45.5%) followed by service responsiveness (38.6%), and communication of supporting staff with users (25%) [2,5,7,8,10,13-17,19,21-23,25-27,29,30,32-38,40-43,46,47].

###  Perceived Usefulness

The dimension of perceived usefulness aspects ranges from the impact on productivity, job performance, efficiency, and effectiveness to utility, meeting the user’s expectations and needs, facilitating work, saving time, solving business problems, and accelerating work accomplishment. Among these factors, impact on job performance (25%) and meeting users’ needs and expectations (18.2%) were the most highly appeared items in the models [7,8,10,12,16,18,23,27,29-31,33,34,39,41-43,45,46].

###  System Use

Factors considered in this dimension include user work dependency on the information system, diversity of the system usage, time spent on working with the system, frequency and volume of the usage, and integration of the system use into the routine workflow. The three most prevalent factors in the models were the usage frequency and volume (13.6%), time spent on working with the system (6.8%), and user’s work dependency on the information system (6.8%)[8-10,13,15,18,20,33].

###  User Characteristics

There are nine attributes identified in the dimension of user characteristics. These factors could be enlisted as follows: user age, gender, attitude, expectation level, experience, anxiety, skills, current working unit, and understanding of the system. User skills (18.2%), user expectations (18.2%), and user experience (13.6%) were the highly mentioned factors that are related to the user satisfaction with information system in user characteristics dimension [5,8,15-18,21-23,25,27,29,30,33,36-38,42,43].

###  Organizational Structure and Management Style

Organizational structure and management style were among dimensions related to user satisfaction with information systems. This dimension included the culture of user involvement in the system development, top management involvement and support, the existence of uniform procedures, style of scheduling works, task assignment style, organizational task structure, and managers’ behavior with users. Among these items, the culture of user involvement (22.7%) and top management involvement and support (22.7%) were highly addressed factors among models of the user satisfaction models [5,6,8,14,17,19,21-23,27,28,30,36,43].

## Discussion

Information quality is of utmost importance in health systems, as it is essential for quality decision-making [48]. Information quality has different aspects ranging from information availability to information understandability [49]. Any issue in the quality of received information can compromise outcomes and can be fatal in some processes such as patient care. Evidence shows that information quality influences the information system use and consequently, the user satisfaction with the system [50]. The primary users of information in Hospital Information Systems (HISs) are clinicians whose first aim is to make an informed decision toward providing the best possible health care to their patients, and restoring patient health. HISs that have proven to help clinicians achieve this goal can increase their satisfaction with the system. The secondary users such as administrators, managers, insurers, quality auditors, accreditation bodies, and policymakers were satisfied with the system if the system provided high-quality information to be used for better management of health system and better evidence-based policymaking in health system toward strengthening health system [51]. Information quality was also of importance for researchers. All attributes identified for information quality in the reviewed studies can contribute to user satisfaction with HIS. The higher the quality of information residing in HIS, the more valid the research result, and the more satisfied they are with the system. Utilizing key enablers such as policies [51,52] and governance for improving information quality in HIS can lead to an increase in the users’ satisfaction with the system. Some reported that appropriate information modeling could enhance information quality [52,53]. System quality has been referred to as engineering-oriented attributes of the system and user experience from a technical, design, and operational perspective [54]. System quality is the measure of the contribution of the information system to the organization [55]. This dimension has been found to be related to user satisfaction with HIS [56]. Some referred to system quality, as system performance. The higher the system quality, the higher the user satisfaction [50]. Evidence implies that the clinical information system performance is among the significant determinants of clinicians’ satisfaction with the system [2,57]. Service or vendor support quality influences the users’ satisfaction through meeting their needs during the implementation phase of the system [50]. Vendor support quality of HIS, as well as clinical information system, has been found to be strongly correlated with user satisfaction with the system [33,58]. The negative impact of low vendor support quality has been reported on the users’ perception of the system quality and, accordingly, their satisfaction with the system [50]. HIS users are often unfamiliar with the technical aspects of the system, and an appropriate technical service must be available to them whenever needed [4]. Perceived usefulness was considered one of the main dimensions of user satisfaction with HIS. The notion of perceived usefulness implies that users have the willingness to use information systems based on the degree that they believed it would assist them in performing their job [59]. The result of one study confirms that perceived usefulness can affect nurses’ satisfaction with the information system [60]. Perceived usefulness has been suggested to be associated with the system capability [61]. Some believe that perceived usefulness cannot be separated from the context in which the information system is implemented. For instance, inappropriate infrastructure, which results in interrupted power supply, can have negative effects on perceived usefulness and accordingly results in the low user satisfaction with the information system [59]. Users can not get satisfaction or dissatisfaction with the information systems if they do not use the system [13]. User satisfaction can reflect the result of assessing the value of information system by the user through comparing its benefits or rewards against its related efforts or costs [62]. System use is related to user satisfaction through having an impact on the user job performance [10]. This can be attributed to the fact that the more use of the system leads to better performance with that system [20]. The association between user satisfaction with the clinical information system and the system use has been reported [33,63]. Evidence shows interdependency between clinical information systems use dependency among nurses and their satisfaction with the system [15]. Individual differences are important aspects of examining human behavior and interactions. User characteristics such as age, gender, education, organizational level, familiarity with the search topic (domain expertise), motivation, experience, computing anxiety, and skills can influence the user satisfaction with the information system [17,21,22,64]. This impact has also been reported about user satisfaction with electronic health record systems. Characteristics such as practice size and type of user are among the features reported to be associated with the user satisfaction with the system [63]. Organizational structure and management style provides the context for the use of HISs [21]. The organization with participatory management styles can influence user satisfaction with information systems. If the organization structure and management style could facilitate the utilization of information systems by users, they will be more satisfied with computing with the systems [22].

## Conclusion

 User satisfaction with HISs is a multi-dimensional construct that is associated with various factors. These factors range from individual characteristics of the user to the organizational context, management style, technical quality and performance of the system itself, vendor support quality, and the system’s perceived usefulness to the system use. If all these factors are adequately considered in the process of developing, designing, implementing or purchasing information systems, the higher user satisfaction with the system will be likely.

## Acknowledgement

 This paper is based on MSc thesis in Health Information Technology at Tabriz University of Medical Science.

## Conflict of Interest

 The authors have no conflicts of interest to disclose.
